# Postpartum HPV Vaccination Rate and Differences in Background Characteristics Between HPV Vaccinated and Unvaccinated Postpartum Women: Strict Monitoring and Follow-Up of Postpartum HPV Vaccination Program

**DOI:** 10.3389/fimmu.2021.626582

**Published:** 2021-05-12

**Authors:** Chung-Yuan Lee, Chih-Jen Tseng, Chi-Chang Chang, Meng-Chih Lee, Shun-Fa Yang

**Affiliations:** ^1^ Institute of Medicine, Chung Shan Medical University, Taichung, Taiwan; ^2^ Department of Obstetrics and Gynecology, Chiayi Chang Gung Memorial Hospital, Chiayi, Taiwan; ^3^ Department of Nursing, Chang Gung University of Science and Technology, Chiayi, Taiwan; ^4^ Department of Obstetrics and Gynecology, Chung Shang Medical University Hospital, Taichung, Taiwan; ^5^ Department of Obstetrics and Gynecology, E-Da Hospital, Kaohsiung, Taiwan; ^6^ Department of Medical Research, Chung Shan Medical University Hospital, Taichung, Taiwan

**Keywords:** postpartum period, uterine cervical neoplasms, vaccination, human papillomavirus (HPV), papillomavirus vaccines

## Abstract

There is a need to increase the vaccine completion rates in women who have already received human papillomavirus (HPV) vaccines. With vaccines requiring multiple doses, designing a vaccination control program and increasing the proportion of women who complete vaccination are critical and remain as huge challenges. Currently, there are no published reports on the differences in the background characteristics between postpartum women who are vaccinated or unvaccinated against HPV. This study aimed to determine the vaccination rates of the second and third doses of HPV vaccination utilizing an achievable HPV vaccination program in postpartum women. In this retrospective study, 243 postpartum women attending Chiayi Chang Gung Memorial Hospital between March and September 2014 were enrolled. These women were classified into two groups: one group received the HPV vaccine under a practical, controlled postpartum HPV vaccination program, and the other group did not. The rates for the second and third rounds of HPV vaccination in postpartum women were calculated. The differences in the background characteristics between the two groups were determined using the Student’s t test, chi-square test or Fisher’s exact test, and the multiple logistic models, as appropriate. Under the controlled postpartum HPV vaccination program, the completion rate for the three doses of postpartum HPV vaccination was 97.2%. Significant differences were observed according to maternal age, gender of the newborn, and postpartum Pap smear results between the two groups in our study. In conclusion, the controlled postpartum HPV vaccination program is a reasonable method for achieving an excellent completion rate for the three doses of postpartum HPV vaccination and may be a good model for any multiple-dose vaccination protocol.

## Introduction

Cervical cancer in women is the greatest disease burden caused by the human papillomavirus (HPV) infection (1). The estimated age-standardized incidence of cervical cancer of 13.1 per 100 000 women globally, varied widely among countries, with rates ranging from less than 2 to 75 per 100 000 women (2). However, associations between pregnancy and HPV infection and the trend of increasing cervical cancer risk with greater number of live births are controversial (3–5). Cervical ectropion in women of child-bearing age (6, 7), thinning of the cervical epithelium during pregnancy, and injuries to the cervix during vaginal delivery may increase the risk of HPV infection in postpartum women (8). Moreover, women with multiple vaginal deliveries and those of young age at their first full-term delivery have a higher risk of cervical cancer (9). Therefore, HPV vaccination is still advantageous for postpartum women. Studies have shown that postpartum HPV vaccination is safe in the postpartum period and in lactating women (10, 11). Considering that postpartum women are more susceptible to HPV infection, and a scheduled postpartum visit can increase the convenience and acceptance of postpartum women toward HPV vaccination, the American Congress of Obstetricians and Gynecologists (ACOG) recommends postpartum HPV vaccination (12). The Taiwan Society of Perinatology and the Taiwan Maternal Fetal Medicine Society also promoted postpartum HPV vaccination in 2013 and 2014, respectively.

Currently, commercially available HPV vaccines include bivalent HPV vaccine (vaccination schedules at 0, 1, and 6 months) (13–15); the quadrivalent vaccine; and the 9-valent vaccine (both vaccinations are scheduled at 0, 2, and 6 months) (16–21); all require three doses after 15 years of age. All clinical data on women who have completed HPV vaccination showed reductions in the incidence of cervical precancerous lesions and cervical cancer (14–19, 22, 23). HPV vaccination also provided satisfactory protection against oncogenic HPV subtypes (14, 15, 19, 22, 23).

According to ACOG data, in the US, although 50% of adolescent girls aged 13-17 years received at least one dose of HPV vaccine, only 33% completed the required three doses (24). A study on postpartum women receiving HPV vaccination in the US showed that 98.6% of postpartum women felt that HPV vaccination during postpartum visits is convenient and worthwhile (25). Although most postpartum women had a high acceptance of this vaccination program, only 30.7% completed the three doses.

Clinical observations have shown that without effective monitoring and management of HPV vaccination, the proportion of women receiving the three doses of HPV vaccine may decrease (25–28). Studies on the quadrivalent HPV vaccine in Australia (29) and the bivalent HPV vaccine in Scotland (30) showed that uncompleted vaccination regimens with fewer than three doses of HPV vaccine results in significantly decreased protection. To date, alongside increasing Pap smear coverage (31–37) and accuracy (38) for cervical cancer prevention, comprehensive promotion of HPV vaccines (10), and increasing HPV vaccination rates in women are considered effective (39, 40). Most important is the need to increase the vaccine completion rate in women who have already received HPV vaccines. Therefore, with vaccines requiring multiple doses, designing a vaccination control program, and increasing the proportion of women who complete vaccinations are critical and remain huge challenges.

Furthermore, to date, there have been no reports examining the differences in background characteristics between postpartum women who have been vaccinated against HPV and those who have not been vaccinated. In this retrospective study, we provided a practical method and model for the promotion and improvement of the coverage rates of the second and third doses of HPV vaccination. Therefore, this study aimed to determine the vaccination rates of the second and third doses of HPV vaccination utilizing a reasonable HPV vaccination program in postpartum women.

## Materials and Methods

We studied the rationale for postpartum HPV vaccination program in our hospital. In this retrospective study, a total of 243 postpartum women, delivered by a single designated attending physician (Chung-Yuan Lee, MD, PhD) from the Department of Obstetrics and Gynecology in Chiayi Chang Gung Memorial Hospital, from March to September 2014 were enrolled. These women were classified into two groups: one group received the HPV vaccine under an achievable, controlled postpartum HPV vaccination program, and the other group did not. The attending physician designed the postpartum HPV vaccination program and performed the delivery for most of the women attending our hospital, including all study subjects. We examined the postpartum HPV vaccination and completion rates for the three doses of HPV vaccines under effective monitoring and determined the differences in background characteristics between vaccinated and unvaccinated women. We also recorded patients’ characteristics such as maternal basic data including age, education level, blood type, occupation, religion, race, parity, postpartum sterilization, Group B *Streptococcus* (GBS) infection, gestational age, delivery method, single/twins, prenatal testing selection, pregnancy status, delivery complication, and postpartum Pap smear result. The characteristics of the newborn including gender and newborn outcomes were also recorded. This study was approved by the Institutional Review Board (IRB) of the Chang Gung Medical Foundation for clinical trials, and the need for informed consent was waived (IRB No.: 201700098B0).

The inclusion criteria for this study were as follows: delivery cases by a single designated attending physician from the Department of Obstetrics and Gynecology in Chiayi Chang Gung Memorial Hospital, postpartum women (with and without maternal delivery complications and regardless of premature, full-term, or post-term delivery), and live births (with and without newborn complications). To ensure the quality and content of explanations, the designated attending physician (Chung-Yuan Lee, MD, PhD), with 10 years of experience in the field of HPV vaccination, who designed the postpartum HPV vaccination program, provided a standard explanation on the benefit, cost, and schedule of postpartum HPV vaccination, as well as vaccine efficacy to the study subjects during the postpartum hospitalization period. The exclusion criteria were the following: non-postpartum women, stillbirths, delivery cases not managed by the designated attending physician, and subjects who were unable to receive the standard explanation of the designated physician in person. Any questions on HPV vaccination asked by the postpartum women were answered. All postpartum women who received HPV vaccine understood the indications for HPV vaccination.

Subjects who agreed to undergo vaccination were administered the first dose (the date of the first HPV vaccine dose was also the date of the first postpartum follow-up visit) during the postpartum hospitalization period. The follow-up visit for postpartum examination was arranged at discharge. The contact phone numbers of included subjects had already been recorded in the outpatient records (mobile phone numbers of both spouses or any telephone number for contact) at the time of the prenatal oral glucose tolerance test (OGTT) at 24-28 weeks of gestation.

**Figure 1:**
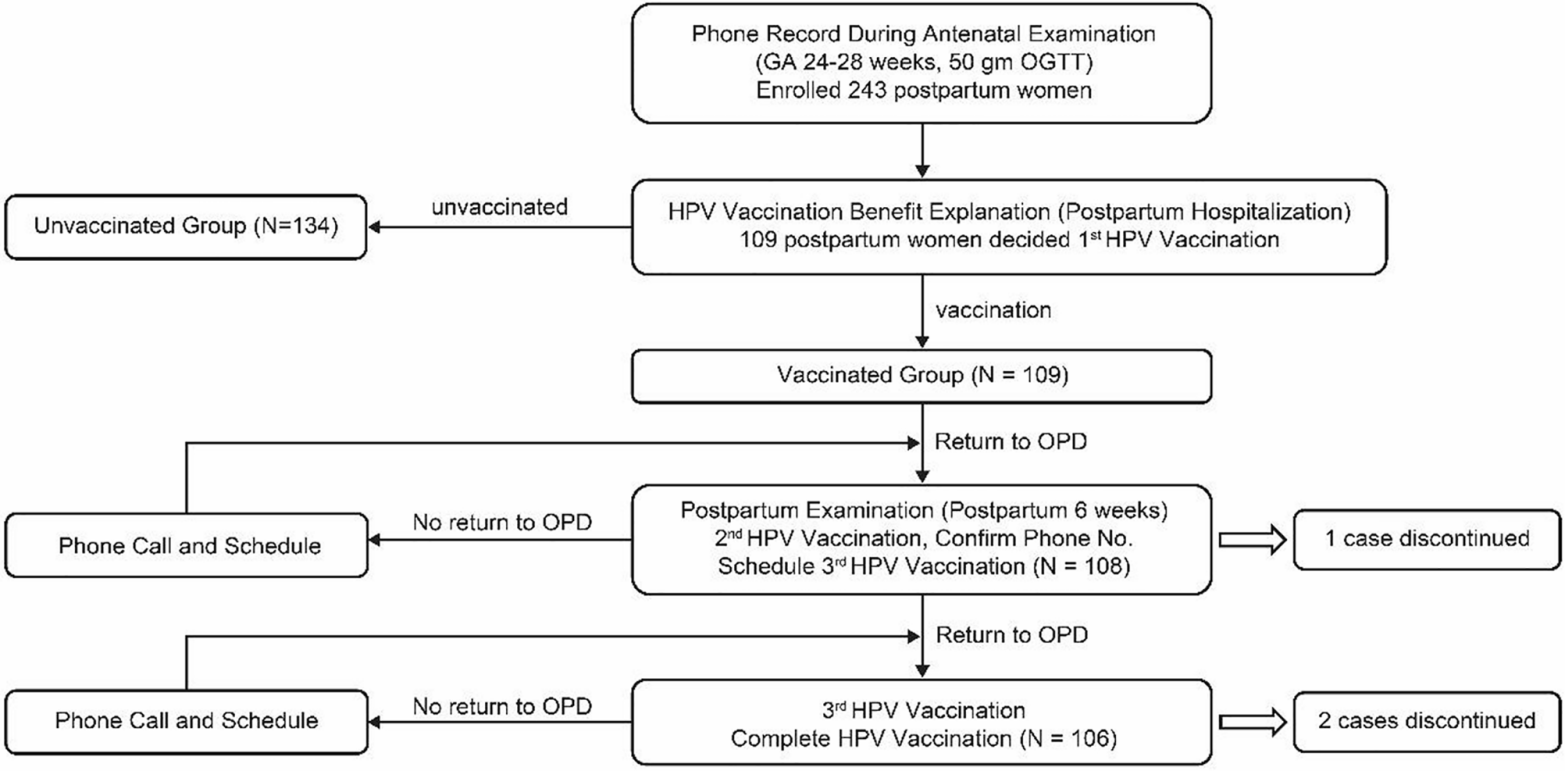
Flow chart of subjects in the controlled postpartum HPV vaccination program. GA, gestational age; OGTT, oral glucose tolerance test; HPV, human papillomavirus; OPD, outpatient department.

The second HPV vaccine dose and a reconfirmation of the subject’s contact phone number were carried out at the outpatient postpartum visit. When the date of the third HPV vaccine dose was arranged, the subject was asked to set a reminder on their mobile phones or other devices for the follow-up visit. If patients did not automatically return for a follow-up visit after HPV vaccination, the subjects were contacted for a follow-up visit and an appointment was automatically made. If the subject still did not return for a follow-up visit, a maximum of three repeated attempts was made to contact her. **Figure 1** outlines the postpartum HPV vaccination program. We used the bivalent HPV vaccine (vaccination schedules at 0, 1, and 6 months) and the quadrivalent vaccine (vaccination schedules at 0, 2, and 6 months) as the postpartum vaccines. The cost for both the bivalent HPV vaccine and the quadrivalent vaccine are the same (114 USD) in our hospital. Patients personally pay for the HPV vaccination. We left it to the postpartum women to decide their choice to be vaccinated or not.

### Covariate

We collected maternal and newborn related variables from the medical records. The maternal variables included age, education, blood type, occupation, religion, race, parity, abdominal tubal sterilization (ATS), infection with group B *Streptococcus* (GBS), gestational age, delivery method (classified as normal spontaneous vaginal delivery and cesarean section), single birth or not, selection of Down screening (classified as quadruple test, amniocentesis, NIPT [noninvasive prenatal test], no screening), pregnancy status (defined as normal and high risk; high risk pregnancy including gestational diabetes mellitus [GDM], pregnancy induced hypertension, preeclampsia, hyperthyroidism, vacuum delivery/dystocia, fetal arrhythmia during antenatal stage, preterm labor, intrauterine growth retardation [IUGR], low birth weight, precipitated labor), complication (defined as postpartum hemorrhage [PPH], third–fourth degree perineal laceration, meconium stain 2+-3+, maternal fever), postpartum Pap smear result (classified as normal, abnormal and no Pap smear; abnormal Pap smear including inflammation, atypical squamous cell of undetermined significance [ASCUS], cervical intraepithelial neoplasia grade 1 [CIN1], grade 2 [CIN2], grade 3 [CIN3]). The variables of the newborn included gender, neonatal care unit (including baby room, IMU [intermediate neonatal care unit] and PNICU [pediatric neonatal intensive care unit]), APGAR score at 1 min/5 min.

### Statistical Analysis

For the statistical analysis, the continuous variables were analyzed using the Student’s t test; the categorical variables were analyzed using the chi-squared test or Fisher’s exact test, as appropriate. Furthermore, we selected the significant related covariates of vaccination willingness in the multivariate analysis. The multiple logistic models were used to determine the effect factors for vaccination willingness. Data were presented as odds ratio (OR) and 95% confidence interval (CI). A two-sided *P*-value <0.05 was considered statistically significant. IBM SPSS for Windows version 22 (IBM Corp., Armonk, N.Y., USA) was used for data analysis.

## Results

This study included a total of 243 women whose deliveries were assisted by a designated single attending physician from March to September 2014. Overall, 109 (44.9%) women were administered postpartum HPV vaccination and 134 (55.1%) were not. During the study period, the postpartum HPV vaccination program (**Figure 1**) in our hospital was used for the strict monitoring and follow-up of postpartum HPV vaccination cases. In the postpartum HPV vaccination group, all (100.0%, N=109) women were vaccinated with the first dose. The completion rates for the second and third postpartum HPV vaccine doses were 99.1%, (N=108) and 97.2% (N=106), respectively. During the study period, the six patients who did not follow the schedule for postpartum HPV vaccines were all contacted telephonically. Three subjects completed the three postpartum HPV vaccines doses outside our hospital. Three subjects did not complete the postpartum HPV vaccinations doses (one did not complete the second and third doses, and two did not complete the third dose) for the following reasons: busy schedules, long-term residence in mainland China, and unknown reasons.

We further examined the background characteristics of differences between vaccinated and unvaccinated subjects (**Table 1**). Significant differences were found in age between the vaccinated (peak: 31-35 years) and unvaccinated (peak: 26-30 years) groups (*P=*0.02). There were significant differences in educational levels of the women between the two groups (*P=*0.02). The proportion of women with university-level education and above was higher in the vaccinated group than in the unvaccinated group, whereas the proportion of women with below university-level education was higher in the unvaccinated group than in the vaccinated group.

**Table 1:** Characteristics of maternal and newborn between vaccinated and unvaccinated groups.

	Vaccinated group		Unvaccinated group		*P*-value
	number	%	number	%	
Maternal	109		134		
Age (years)	32.3 ± 4.7		30.5 ± 4.3		0.002
16-20	0	0	2	1.5	0.023
21-25	7	6.4	13	9.7	
26-30	31	28.4	53	39.6	
31-35	41	37.6	47	35.1	
36-40	26	23.9	19	14.2	
41-45	4	3.7	0	0	
Education					
University/graduate school	64	58.7	53	39.6	0.018
Junior college	16	14.7	22	16.4	
Senior high school	25	22.9	48	35.8	
Junior high school/primary school	4	3.7	11	8.2	
Blood Type					
O	47	43.5	60	45.8	0.822
A	29	26.9	39	29.8	
B	21	19.4	22	16.8	
AB	11	10.2	10	7.6	
Unknown	1		3		
Occupation					
Medical staff	24	22.0	23	17.2	0.031
Civil servants	14	12.8	8	6.0	
Service industry	23	21.1	30	22.4	
Laborer	1	0.9	11	8.2	
Unemployed	47	43.1	62	46.3	
Religion					
Christianity	3	2.8	1	0.7	0.514
Taoism	11	10.1	11	8.2	
Buddhism	7	6.4	8	6.0	
N/A	88	80.7	114	85.1	
Race					
Taiwanese	105	96.3	128	95.5	1
Chinese	3	2.8	4	3.0	
Vietnamese	1	0.9	2	1.5	
Parity					
Primipara	54	49.5	59	44.0	0.392
Multipara	55	50.5	75	56.0	
ATS					
+	6	5.5	8	6.0	0.877
−	103	94.5	126	94.0	
GBS infection					
+	23	21.1	25	18.7	0.675
−	78	71.6	102	76.1	
No culture	8	7.3	7	5.2	
Gestational age (weeks)^*^,†^^					
≥42 0/7	0	0	0	0	0.918
37 0/7-41 6/7	102	93.6	124	92.5	
34 0/7-36 6/7	5	4.6	8	6.0	
≤33 6/7	2	1.8	2	1.5	
Delivery method					
NSD	89	81.7	107	79.9	0.724
C/S	20	18.3	27	20.1	
Single birth					
Yes	107	98.2	133	99.3	0.589
No	2	1.8	1	0.7	
Down screening^‡,¶^					
Quadruple test	64	58.7	88	65.7	0.251
Amniocentesis	37	33.9	37	27.6	
NIPT	6	5.5	3	2.2	
No screening	2	1.8	6	4.5	
Pregnancy status					
Normal	93	85.3	111	82.8	0.600
High risk	16	14.7	23	17.2	
Complication					
Yes	11	10.1	9	6.7	0.341
No	98	89.9	125	93.3	
Pap smear result					
Normal	79	72.5	90	67.2	0.006
Abnormal	25	22.9	21	15.7	
No Pap smear	5	4.6	23	17.2	
Newborn	111		135		
Gender					
Male	67	60.4	63	46.7	0.032
Female	44	39.6	72	53.3	
Neonatal care unit					
Baby room	95	85.6	117	86.7	0.411
IMU	10	9.0	15	11.1	
PNICU	6	5.4	3	2.2	
APGAR score at 1 min					
≥7	111	100	133	98.5	0.503
<7	0	0	2	1.5	
APGAR score at 5 min					
≥7	111	100	135	100	–
<7	0	0	0	0	

^*^Comparison of gestational age ≥37 (0/7) weeks and <37 (0/7) weeks between vaccinated and unvaccinated groups: P=0.752.

^†^Gestational weeks ≥42 (0/7): Post term; 37 (0/7)-41 (6/7): term; 34 (0/7)-36 (6/7): mild preterm; ≤33 (6/7): severe preterm.

^‡^Comparison of NIPT with quadruple test between vaccinated and unvaccinated groups: P=0.179.

^¶^Comparison of amniocentesis with quadruple test, NIPT, and no screening between vaccinated and unvaccinated groups: P=0.286.

ATS, Abdominal tubal sterilization.

GBS, Group B Streptococcus.

NSD, Normal spontaneous vaginal delivery.

C/S, Cesarean section.

NIPT, Noninvasive prenatal test.

APGAR score, Including appearance, pulse, grimace, activity, and respiration score related to neonatal outcomes.

IMU, Intermediate neonatal care unit.

PNICU, Pediatric neonatal intensive care unit.

There were significant differences in occupation between the vaccinated and unvaccinated groups (*P=*0.03). A higher proportion of vaccinated subjects were medical staff or civil servants, while a higher proportion of unvaccinated subjects were in the service industry, laborers, or unemployed. There were significant differences in postpartum Pap smear results between the vaccinated and unvaccinated groups (*P=*0.006). Of the 109 subjects in the vaccinated group 72,5% (N=79), 22.9% (N=25), and 4.6% (N=5) had normal or abnormal results, or did not undergo Pap smears, respectively. All subjects in the vaccinated group with abnormal Pap smear results completed the three doses of HPV vaccine. Among the three subjects who did not complete three doses of HPV vaccines, all had normal postpartum Pap smear results. There were significant differences in the gender of the newborn (*P=*0.03).

There were no significant differences in maternal blood type (*P=*0.84), religion (*P=*0.51), race (*P=*1.00), primipara/multipara, postpartum sterilization, and infection with group B *Streptococcus* during pregnancy between the two groups (*P=*0.39, 0.88, and 0.68, respectively). No significant differences were observed in post-term deliveries (≥42 0/7 weeks), full-term deliveries (37 0/7-41 6/7 weeks), mild preterm deliveries (34 0/7-36 6/7 weeks), and severe preterm deliveries (≤33 6/7) between the two groups (*P=*0.92); gestational periods ≥37 0/7 and <37 0/7 (*P=*0.75); delivery method and delivery of single/twin births (*P=*0.72 and 0.59, respectively). There were no significant differences between the vaccinated and unvaccinated groups in selection of prenatal Down syndrome screening (*P=*0.25). Further comparisons between NIPT and quadruple test, and between amniocentesis with non-invasive Down syndrome screening (NIPT, quadruple testing, and no screening) also showed no statistical differences (*P=*0.18 and 0.29, respectively). There were no significant differences between the vaccinated and unvaccinated groups in terms of normal/high-risk pregnancies or in complications during delivery (*P=*0.60 and 0.34, respectively).

In neonatal outcomes, there were no significant differences between the vaccinated and unvaccinated groups in neonatal care unit (*P=*0.41), in Appearance, Pulse, Grimace, Activity, and Respiration (APGAR) score (≥7 and <7 points) in the first and fifth minute (*P=*0.50).

The effect factors for vaccination willingness using the multiple logistic regression is shown in **Table 2**. By age in model 1, for each additional year, there was 9% increase in the willingness to receive postpartum HPV vaccination (95% CI: 1.02-1.17). For occupation, laborers were 90% (95% CI: 0.01-0.90) significantly less willing to receive HPV vaccine than medical staff in model 1. According to model 1 analysis, women who did not have a Pap smear test were 75% (95% CI: 0.09-0.71) significantly less likely to receive HPV vaccine than those who had a normal Pap smear result. After eliminating the non-significant variables (education and occupation) in model 1, the results of age and postpartum Pap smear test were similar to those of the initial model 1, and the impact of gender of the newborn became significant. A mother who gave birth to a male was 1.71 times more likely to receive HPV vaccine than a mother who gave birth to a female in model 2.

**Table 2:** The effect factors of vaccination willingness in the multiple logistic regression analysis.

Variable	Model 1			Model 2		
	OR	95% CI	*P*-value	OR	95% CI	*P*-value
Intercept	0.01		0.002	0.06		0.003
Age	1.09	(1.02 - 1.17)	0.007	1.08	(1.02 - 1.15)	0.009
Education level			0.248			
University/graduate school	3.43	(0.93 - 12.73)	0.065			
Junior college	2.23	(0.55 - 8.99)	0.260			
Senior high school	2.18	(0.59 - 8.11)	0.246			
Junior high school/primary school	1					
Occupation			0.213			
Civil servants	1.38	(0.47 - 4.11)	0.558			
Service industry	1.08	(0.45 - 2.60)	0.862			
Laborer	0.10	(0.01 - 0.90)	0.040			
Unemployed	1.26	(0.55 - 2.87)	0.588			
Medical staff	1					
Pap smear			0.015			0.015
Normal	1			1		
Abnormal	1.38	(0.68 - 2.79)	0.368	1.35	(0.69 - 2.65)	0.378
No Pap Smear	0.25	(0.09 - 0.71)	0.009	0.25	(0.09 - 0.71)	0.009
Newborn Gender						
Male	1.59	(0.91 - 2.80)	0.105	1.71	(1.002 - 2.92)	0.049
Female	1			1		

OR, odds ratio; CI, Confidence Interval.

## Discussion

This study employed a postpartum HPV vaccination program designed by our hospital for the strict monitoring and follow-up of postpartum HPV vaccination cases. We retrospectively analyzed the data of 243 postpartum women who were or were not vaccinated against HPV. We evaluated the completion rate for the second and third HPV vaccine doses under effective monitoring. We also assessed the differences in background characteristics between vaccinated and unvaccinated women.

We found that the completion rates for the second and third HPV doses were not inferior to those of the global 9-valent HPV vaccine clinical trial (19) and the 2015 Australian publicly funded HPV vaccination program (41). Our study data clearly revealed superior completion rates to those of HPV vaccination in another hospital of similar scale in southern Taiwan (data from E-Da Hospital, Kaohsiung, Taiwan; unpublished results) during the same period. This could be because that hospital had no HPV vaccination program and was unable to effectively monitor or follow-up on women receiving HPV vaccination.

It was especially surprising to find no difference between groups with respect to infection with Group B *Streptococcus*, preterm delivery, poor birth status of newborns, and admission of newborns into the intensive care unit, high-risk pregnant women, delivery complications, and pain after cesarean section (**Table 1**). Generally, the anxiety felt by pregnant women regarding Group B *Streptococcus* infection during pregnancy, preterm delivery, twin pregnancy, poor birth status of newborns, and admission of newborns into the intensive care unit might cause women to be more focused on neonatal outcomes. Thus, postpartum women may ignore the importance of postpartum HPV vaccination, affecting the proportion of women who were willing to receive HPV vaccination. The findings seem to suggest that these variables do not affect the decision for postpartum HPV vaccination. Further analysis of gestational age at delivery ≥37 0/7 and <37 0/7 revealed no significant differences between the vaccinated and unvaccinated groups, which suggests that the experience of postpartum women with premature babies might not affect their willingness to receive postpartum HPV vaccination. A cross-analysis of the costs of prenatal screening for Down syndrome revealed no significant differences between vaccinated and unvaccinated women in those who received the relatively cheaper quadruple test (cost, 81 USD) when compared to those who received the more expensive NIPT, (massively parallel shotgun sequencing [MPSS]-based NIPT [486.5 USD] and single nucleotide polymorphism [SNP]-based NIPT [778.5 - 1232.6 USD]) (*P=*0.18), suggesting that the choice of Down syndrome screening does not play a major role in their acceptance of postpartum HPV vaccination.

Multiple logistic regression was used to assess the differences in the background characteristics between vaccinated and unvaccinated subjects (**Table 2**), we observed significant differences in age between the groups. A possible reason may be that older postpartum women are more concerned about their health. Postpartum women with male newborns were more determined to receive postpartum HPV vaccination, which might relate to the traditional importance attached to the birth of a male child by the Chinese and compensate the postpartum women’s hard work with joy. Even though, there is no significant difference in education level and occupation (*P=*0.25 and 0.21, respectively) (in **Table 2**), a greater proportion of medical staff postpartum women tended to receive postpartum HPV vaccination than laborer postpartum women, probably because they had a better understanding of the benefits of postpartum HPV vaccination, valued health more, and were better off financially. Although no postpartum Pap smear was carried out in postpartum women receiving the first dose of HPV vaccination, an analysis of postpartum Pap smears found significant differences between the vaccinated and unvaccinated groups (*P=*0.02) (**Table 2**). Subjects in the vaccination group with abnormal Pap smears all successfully completed the three doses of HPV vaccine, possibly because they required Pap smear follow-up and were also aware of the cervical protection benefits of HPV vaccination.

The limitations of the study were, first, that it was a single-center study with a limited sample size. Nonetheless, we believe that our designed postpartum HPV vaccination program is achievable and has the potential for positive outcomes for all postpartum women who are willing to receive HPV vaccination under strict monitoring. Furthermore, we did not exclude postpartum women who had received HPV vaccine before delivery from the unvaccinated group. In fact, the HPV vaccination rate among those aged 18-55 years is only about 4% in Taiwan. This does not seem to have influenced the analysis and could truly reflect the differences in background characteristics between vaccinated and unvaccinated postpartum women.

## Conclusion

The study comprehensively examined differences in background characteristics between HPV vaccinated and unvaccinated postpartum women. This study found that age, gender of the newborn, and postpartum Pap smear results were factors determining postpartum HPV vaccination in postpartum women. We found much improved HPV vaccine completion rates when strict monitoring and follow-up of postpartum HPV vaccinations in women were ensured according to the postpartum HPV vaccination program at our hospital. Therefore, the postpartum HPV vaccination program formulated by our hospital can be used as a reference to improve the HPV vaccine completion rates in similar settings. This may be further extended to similar vaccines that require multiple injections to effectively increase vaccine completion rates and, in turn, protection.

In the next phase, we plan to study the satisfaction of postpartum women who received HPV vaccination in Taiwan, including analysis of reasons why postpartum women did not accept the HPV vaccine. This could serve as a reference for the policy to publicly fund postpartum women HPV vaccination programs. Future studies should also include the follow-up of Pap smear abnormalities, local cervical therapy (cervix cryotherapy, electrocauterization, and conization) as well as HPV infection status according to the database of our postpartum HPV vaccination program, to evaluate the efficacy of postpartum HPV vaccination.

## Data Availability Statement

The original contributions presented in the study are included in the article/supplementary material. Further inquiries can be directed to the corresponding author.

## Ethics Statement

Written informed consent was obtained from the individual(s) for the publication of any potentially identifiable images or data included in this article.

## Author Contributions

C-YL designed the study. C-YL conducted the systematic review and analyzed the data with help from C-CC. C-YL wrote the manuscript, and then C-JT, M-CL, and S-FY critically revised it before all authors read and approved the final version. All authors contributed to the article and approved the submitted version.

## Conflict of Interest

The authors declare that the research was conducted in the absence of any commercial or financial relationships that could be construed as a potential conflict of interest.
